# What credentials are required for robotic‐assisted surgery in reconstructive and functional urology?

**DOI:** 10.1002/bco2.238

**Published:** 2023-04-27

**Authors:** Frances Harley, Eva Fong, Henry Han‐I Yao, Hashim Hashim, Helen E. O'Connell

**Affiliations:** ^1^ Department of Surgery University of Melbourne Melbourne Victoria Australia; ^2^ Department of Urology Urology Institute Auckland New Zealand; ^3^ Bristol Urological Institute Southmead Hospital, North Bristol NHS Trust Bristol UK; ^4^ Department of Epidemiology and Preventive Medicine Monash University Melbourne Victoria Australia

**Keywords:** credentialing, reconstructive and functional, robotic‐assisted surgery, training

## Abstract

**Introduction:**

The increasing popularity of robotic assisted surgery (RAS) as it is implemented in to sub specialities poses many challenges to ensuring standards in quality and safety. The area of Reconstructive and Functional Urology (RFU) has a wide range and largely complex heterogeneous procedures. In recent years RFU has started to incorporate RAS as the primary method to undertake these procedures due to improved vision, dexterity, and access to deep cavities. To ensure patient safety majority of institutions maintain minimal requirements to operate using RAS however across specialities and institutions these greatly vary.

**Methods:**

A narrative review of all the relevant papers known to the author was conducted.

**Results:**

Specific challenges facing RFU is the inability to rely on case numbers as a surrogate means to measure competency as well the ongoing consideration of how to differentiate between surgeons with robotic training and those with the clinical experience specific to RFU.

**Conclusion:**

This review explores current models of training and credentialling and assess how it can be adapted to suggest a standardised guideline for RFU to ensure the highest standards of patient care.

## 1 INTRODUCTION

Robot‐assisted surgery offers multiple advantages including magnified 3D vision, improved manual dexterity, improved access to deep cavities and reduced surgeon fatigue.^1^ Since the introduction of robot‐assisted radical prostatectomy (RARP) in the early 2000s, the use of robot‐assisted surgery in urology and uro‐gynaecology has been expanding including in the field of reconstructive and functional urology (RFU).^1^ Its use is being embraced to improve quality and safety in this diverse field. Because of the improvement in access, for example, to the bladder base and female urethra from within the pelvis, what can be achieved potentially surpasses open surgery.

The recent controversy associated with the adoption and use of surgical mesh, highlights the importance of credentialling and onboarding of surgeons for new procedures or techniques to ensure quality and safety for patients.

Recent reviews have identified variability amongst current policies that are inadequate to ensure surgeon proficiency and therefore protection against patient harm.^2^ As RAS is further implemented, it is widely accepted that standardisation of a detailed credentialling process is required. This area of urology has unique challenges for the credentialling as there is considerable heterogeneity of procedures within this umbrella term.

Broad guidelines have been released by associations such as the British Association of Urological Surgeons (BAUS) on principles for training and credentialling of surgeons. These policies focus on uro‐oncological procedures such as RARP and RAPN (robot‐assisted partial nephrectomy). The Society of European Robotic Gynaecological Surgery (SERGS) has recently addressed the important aspects of credentialling of robotic gynaecology procedures.^3^ The Society published a pilot curriculum in the form of a fellowship‐programme using a standardised modular and stepwise educational programme and validated tests as proof of efficacy similar to BAUS guidelines for RARP and RAPN. These frameworks can be adapted as templates for a proposed curriculum for robot‐assisted surgery in urology and uro‐gynaecology.

In addition to heterogeneity, RFU is challenging to credential because many of the procedures are complex and technically challenging and the case volume for each procedure to be credentialed is low compared to RARP or RAPN. Further the conditions themselves require specialist training to ensure surgical judgement regarding appropriate choice of procedure.

This review explores the training and credentialling process that is described for RAS in other subspecialities to determine aspects that can be adapted to creation of a training and credentialling guideline for reconstructive and functional urology to ensure safe patient care and good outcomes are achieved with ‘onboarding’. What is wrong with the SERGS draft that we are not just adopting it?

## 2 APPLICATION OF RAS IN RECONSTRUCTIVE UROLOGY

### 2.1 Pelvic floor surgery

Meaningful advantages of RAS for intra‐pelvic vaginal wall surgery, such as sacrocolpopexy, is the achievable range of motion, easily targeted magnification and abolition of tremor compared to laparoscope that has resulted in surgeons resorting to the use of tacks (such as ProTack™) and other maneuverers to overcome the challenges of suturing. Robotic surgery for apical vaginal prolapse was first reported in 2006.^4^ Robot‐assisted sacrocolpopexy has been extensively reported and it is now considered the gold standard for apical prolapse. Large meta‐ analysis demonstrated an objective cure rate of 84–100%, a reoperation rate of 3.3% and a recurrence rate of 6.4%.^5^ The RAS approach was found to be associated with increased operating time, but reduced blood loss and length of stay.^5^ It has considerably gained in popularity after the first FDA warning about transvaginal mesh for pelvic organ prolapse in 2008.^6^

Colposuspension has seen a revival in popularity following the transvaginal mesh controversy. There is very limited literature on the RAS Burch Colposuspension. A small pilot study comparing robotic hysterectomy and robotic colposuspension with open hysterectomy and colposuspension demonstrated no difference in continence rates, however there were advantages when comparing blood loss and hospitalisation time.^7^ Tan et al reported on 28 cases of robotic Burch Colposuspension with promising improvements in pad rates and quality of life scores.^8^ As mesh free options become more popular there has been success in several small series using autologous fascia robotic sacral colpopexy. Bock et at showed similar short‐term anatomic outcomes to mesh for women with apical pelvic organ prolapse^9^ and Scott et al reported no intra‐ or postoperative complications, and the median Patient Global Impression of Improvement (PGI‐I) response was 2 (range, 1–3, very much to a little better).^10^

### 2.2 Bladder neck reconstruction and artificial urinary sphincter

Bladder neck contracture, though uncommon, is a challenging complication of prostate surgery and pelvic trauma. A robotic‐assisted approach to V‐Y‐plasty of the bladder neck has recently been described by two small case series by Granieri and Musch et al. The results are largely comparable to open surgery with respect to median surgical time and estimated blood loss.^11^, ^12^ Median length of stay (LOS) varied from 1 to 9.5 days^11^, ^12^ appeared to be shorter compared to one open study that had a median LOS of 13.2 day (SD ± 2.66).^13^

Artificial urinary sphincters have been placed in both males and females using RAS. While there are no randomised comparisons, in females blood loss (17 ml vs. 275 ml), shorter length of stay (3.5 vs. 9.3 days) and intraoperative complication rates (37.5% vs. 62.5%) appear to favour the use of RAS.^14^, ^15^ The use of RAS innovates an established implantation technique with potential significant advantages. However female artificial sphincter is not a common operation and tends to be used in complicated cases rather than as first or second line for severe stress incontinence. In this small series was a high rate of bladder (18%) and vagina (18%) injury reported.^14^ Learning curves play a major role in determining quality and safety, not just the robotic experience but indeed understanding of the anatomy itself and possibly prior experience with low volume procedure even by open surgery.

### 2.3 Upper urinary tract procedures: Pyeloplasty and ureteric reimplantation and reconstruction

Multiple studies^16^, ^17^, ^18^ have demonstrated comparable outcomes with a reduction in operating time for the robotic approach compared to open pyeloplasty surgery, the relative ease of suturing being the key advantage.^1^ Data on RAS ureteric reimplant surgery demonstrates similar complication rates, re‐stricture rates and operative times but with decreased blood loss and length of stay.^19^, ^20^ Compared to conventional open surgery, RAS surgery generally results in reduced wound related morbidity.^21^ Ureteric reconstruction has also utilised RAS and buccal mucosa grafts in a select patient cohort of previously failed pyeloplasty. This small multi‐centre study demonstrated recurrence‐free rate of 95% at 2 years^22^ comparable to both open and laparoscopic rates of conventional re‐do pyeloplasty.^23^ RAS is also being utilised in the paediatric population for re‐do pyeloplasty for recurrent pelvi‐ureteric junction obstruction (PUJO).

### 2.4 Other RAS reconstructive urological procedures

There are several other RAS reconstructive urological procedures described by expert pioneers, generally with case series describing feasibility but in most series with relatively small numbers including: bladder reconstruction and urinary diversion,^24^, ^25^, ^26^ repair of genito‐urinary fistula,^27^ gender affirming neo‐vaginoplasty,^27^ uretero‐enteric anastomic stricture,^27^, ^28^ bladder diverticulectomy,^29^ posterior urethroplasty,^27^ proximal dorsal urethral diverticulectomy^30^ and pelvic mesh removal.^31^

## 3 LESSONS FROM TRAINING OF OTHER RAS PROCEDURES

### 3.1 Learning curves: Pioneers, mentorship and structured modular curriculum

Lessons from urologic and non‐urologic literature demonstrate the learning curves involved in robotic surgery and progress over time from self‐taught pioneers, who then mentored their trainees to structured modular curricula, which are still evolving.

### 3.2 Urologic procedures: RARP

Due to the high volumes of cases, RARP have been established as the entry point of onco‐urology modular training curriculums described by leading organisations such as the European Association of Urology (EAU). An international study by Lovegrove et al reported on a modular pathway for RARP using the ERUS RARP Assessment Score.^32^ They demonstrated that a stepwise approach of increasing difficulty guards against surgeons attempting steps that are too complex and promotes the acquisition of competent operating and patient safety.^32^

### 3.3 Non‐urologic procedures

Rice et al describe the vastly improved learning curves with progression from non‐structured toward structured training from robotic pancreatico‐duodenectomy. From self‐taught pioneers (1st generation) through mentorship alone (2nd generation) to mentorship and modular curriculum, they demonstrated significant reduction in operating times accompanied by a fall in complication rates and blood loss between 1st versus 2nd/3rd generations.^33^ Strikingly, the operating times were lower from the first case for each successive generation: the operating time for 2nd generation was 251.8 min faster than the first generation. Viewed as case load, the starting point for the second generation was greater than 90 cases compared with first‐generation surgeons. The starting point for third‐generation surgeons was greater than 90 cases compared with second‐generation surgeons and greater than 150 cases from first‐generation surgeons.^33^

## 4 NON‐ROBOTIC FUNCTIONAL UROLOGY TRAINING

Case selection requires advanced non‐technical skills: history and physical examination relevant to functional urology problems, interpretation of appropriate urodynamic testing, medical imaging and knowledge of the surgical procedures that have been performed previously. For each procedure, adequate knowledge of the other options and their relative risks and benefits are required.

### 4.1 Robotic training requirements: General

Specific knowledge is required of the risks of a RAS in any given individual due to the need for the extended head down position and the risks of operating in the abdominal cavity. The position of steep Trendelenburg at 25–45 degrees can lead to brain and upper airway oedema in addition to increase in intracranial pressure and cerebral blood flow.^34^ Common risks with positioning have been reported at 0.13% to 3% for Corneal Abrasion, aspiration 6.3%–15%, pressure lesions 35% and airway oedema 0.7%–26%.^35^

The training requirements are tailored to the surgeon's existing skill level, which can be categorised as follows:

- Trainee (novice) surgeon (resident/registrar/fellow)
- Experienced surgeons who have laparoscopic skills
- Experienced surgeons with extensive previous experience of open but minimal laparoscopic experience

Prior to independent operating a stepwise approach is generally recommended including: ‐ completion of a recognised robotic surgical system training course, observership of cases with an experienced surgeon, simulation and wet‐lab training to develop familiarity with the system and cultivate skills^3^, ^36^, ^37^, ^38^ and finally supervised procedures starting with basic procedures before moving to more complex ones.

This process should be done with the demonstration of proficiency and safety in robotic procedural skills as opposed to high numbers alone. In general, a surgeon's proficiency with patients should be complemented by ongoing simulation practice and supervision with the full range of procedures to be undertaken until fully proficient.^3^, ^36^, ^37^, ^38^ Complex or advanced procedures can be undertaken independently once approval has been provided to complete more complex cases.^3^, ^36^, ^37^, ^38^

### 4.2 E‐learning/knowledge and observation

The guidelines recommended by BAUS, NYU and SERGS^3^, ^37^, ^38^ recommend E‐knowledge in addition to sound procedure theory and knowledge of the console. Online training systems are available most commonly from the console provider for example Intuitive (da Vinci) system have an online community providing theoretical training. NYU training specifies this learning should be directed towards covering basic principles of RAS; basic mechanism of the robotic surgical console, surgical cart and 3‐D viewing system; patient selection, preparation, positioning, surgical approaches and techniques, indications and contraindications; port placement location and strategy; procedural steps, surgical cart positioning, intra‐corporeal suturing and knot tying, procedure pre‐planning, system troubleshooting and breakdown.^38^ This is in conjunction with observation of RAS procedures being performed by experts. Emphasis should be on the steps in conducting a safe and effective robotic procedure. This is the approach generally adopted for RARP training elsewhere.

### 4.3 Simulation training

There are several simulation services available: the Robotic Surgical Simulator (RoSS), Mimic ™ Simulation Software and the Simsurgery Educational Platform (SEP), which have proven face, content and construct validity. The simulation aspect should be considered as a step with its own curriculum with scores of >80% in three consecutive tests to be achieved, that is, a competency‐based evaluation. This is juxtaposed with the number of hours on the console, which is less relevant. To ensure simulation practice is effective it should be supervised by an expert surgeon or educationalist. Virtual reality (VR) may also become an increasingly prevalent tool to enhance simulation. A study from Ebbing et al reported their experience of implantation and validation of VR RobotiX Mentor® simulator in RARP modular training. Ebbing's group demonstrating face and content validity for a robotic surgical training programme while focusing on key steps of the surgery such as non‐guided bladder neck dissection (ngBND) and non‐guided neurovascular bundle dissection (ngNVBD).^39^ The group demonstrated face and content validity of both simulator and modules, with construct validity for generic metrics of the ngBND module and generic and task‐specific metrics of the ngNVBD module.^39^ As this technology continues to evolve the introduction of VR training would be largely achievable by most institutions and across subspecialties.

So called ‘wet labs’ where surgical simulation is practices on live animal or cadavers are important to the training progression. These provide a standardised demonstration of a complex range of skills involving placement of ports, troubleshooting of complications in addition to specific operative procedures. Due to cost and access, a wet lab is generally used once full proficiency is achieved with simulation and other training and before independent operating. These ‘wet lab’ training is costly requiring standards of good laboratory practice and complex governance of ethical animal care and donor body systems ongoing work and development would need to be undertaken to ensure the wet‐lab teachings would be of a high standard. To date simulation for RFU procedures is in its early stages. In the future the development of RFU specific simulations requiring virtual or ‘wet lab’ training would be required to demonstrate proficiency.

### 4.4 Credentialing

This requires the establishment of standardised criteria to credential or judge a surgeon's competency. The development and subsequent validation of The Global Evaluative Assessment of Robotic Skills (GEARS) tool was created by deconstructing the fundamental elements of robotic surgical procedures. The six domains evaluated include depth perception, bimanual dexterity, efficiency, autonomy, force‐sensitivity and robotic control. GEARS is the first and currently only such tool, its domains being applicable to any speciality. The tool is applied to a step of the operation that reflects a pivotal point/complex task, which in turn can be applied to various operations‐index or complex to assess competency of a surgeon.^40^ Recently, a study was published applying GEARS to Simulation Model for Robotic Sacrocolpopexy concluding construct and face validity in addition to high interrater reliability.^41^

### 4.5 Modular training

The final aspect of training is applying the theoretical knowledge and skillset to procedures. Modular training breaks down a procedure into key steps/components and entails advancing through the steps of increasing difficulty.^32^ This precedes advancement to operating supervised in theatre with a mentor, eventually with the capacity to independently performing a procedure to a proficient standard.^42^ It is generally expected that each module should be completed prior to progressing to the next step.^3^ Once all modules are completed under supervision, the surgeon or trainee is ready to perform the procedure independently under supervision. Following completion of performing independently for the entirety of the case this will need to be repeated under proctorship—the final step of the credentialing pathway. One of the most well‐known forms of modular training in Urology is the EAU Robotic Urology Section (ERUS) structured curriculum that focuses on RARP.^43^

Recently, there has been publications that works to address establishing performance metrics for the RARP procedure. A study by Mottrie et al developed proficient based progression metrics for RARP that reliably and objectively distinguish experienced and novice robotic surgeons.^44^ The use of errors metrics such as poor vision, instrument clashes and non‐completion of step demonstrated the capacity to discriminate performances. These metrics could be incorporated into the foundation of modular RARP training and in turn be developed for other RAS procedures including RFU.^44^

Specifically, to address the problem of relatively low case numbers in reconstructive and functional urology, one operation can be distributed between trainees (component operating) permitting impactful improvement of technical skills while preserving patient safety^32^ BAUS comments that meeting requirements of learning curve include completing appropriate number of cases in addition to achieving quality indicators appropriate to the procedure such as operative time, estimated blood loss, positive surgical margins, PSA‐ Prostate Specific Antigen, warm ischaemia time, lymph node yield and complication rate in the case of radical prostatectomy.^37^

A key aspect of this model is determining what is a basic or index procedure. Basic operations tend to involve fewer steps with simple dissection while establishing spatial awareness and fine‐tuning purposeful movement. RAS in Urogynaecology/RFU has index operations that are commonly performed laparoscopically such as Burch colposuspension and the authors with robotic experience would propose robotic retropubic sling removal as an index robotic procedure. Both procedures are favourable for their relative predictability permitting standardisation of steps. They are also not rare procedures potentially offering the trainee robotic surgeon the opportunity for repetition to cement and develop skills. Current urology trainees and some surgeons may have some or significant exposure to onco‐urological procedures, such as RARP, but condition specific training in RFU is still required.

Prior to the development of the RAS skills, a model of advanced training, which teaches and evaluates the appropriate clinical evaluation and investigation leading to a decision to treat, as well as a pathway for standardised (or suggested) post‐operative evaluation to ensure that the robotic surgery sits within a whole package of delivering the best possible patient journey. This would give the appropriate balance between technical robotic skills, which are the focus of most current training guidelines and non‐surgical technical skills around clinical history, examination and decision making, which are underrepresented but arguably even more contributory to poor outcomes in reconstructive surgery.^45^

This basic training outline is applicable to any expertise level though would need adjustments based on proficiency. Experienced surgeons in either laparoscopic or open surgery wanting to add RAS to their repertoire should be expected to complete the same curriculum and assessments. For instance, a surgeon may take the equivalent amount of time in E‐knowledge as a resident but progress quickly to Modular training/Proctorship due to their already established technical and procedural skills. Irrespective of experience, each stage requires proof of competency through simulation and proctorship prior to full credentials being granted. Irrespective of a surgeon's robotic skills, it would be expected that a surgeon would have the adequate expertise in either laparoscopic or open surgery if the robot malfunctioned or in a similar practise to laparoscopic surgery, if the conditions became unsafe such as access or life‐threatening bleeding that the operation would be converted to open. It would be an expectation that in addition to robotic training, a surgeon maintains their additional open skillset or have an open surgeon on standby to assist if necessary.

The learning curve is still substantial for an experienced laparoscopic surgeon to transition to robotic surgery. A CUSUM learning curve study of sacrocolpopexy found a learning curve of 78 cases for two surgeons who had each already completed 300 laparoscopic sacrocolpopexies, based on intraoperative bladder/bowel complications.^46^ They had an experienced robotic surgeon supervising for their first two robotic cases. Accordingly, BAUS guidelines 2014 suggest that ‘Mentoring and training must occur in high volume centres by surgeons who have surpassed their “learning curves”’.^37^

## 5 CREDENTIALING

For credentialing, several committees or associations^3^, ^36^, ^37^, ^38^ have provided guidelines for institutions to specify a framework for robotic credentialing across urology and other specialties. Inconsistencies of expectations between institutions and societies potentially allows for inadequate technical proficiency and which may threaten patient safety. A recent Delphi study by Stefanidis et al concluded the importance of standardised robotic surgery credentialing criteria across institutions and have proposed The Fundamentals of Robotic Surgery (FRS), a proficiency‐progression based curriculum for robust acquisition of basic robotic surgery skills.^2^ A framework such as FRS will promote proficiency of robotic surgeons with the potential to positively impact patient outcomes.^2^

1. General requirements^2^, ^35^, ^36^, ^37^ include for credentialing
  * Appropriate local licence with no practice restrictions
  * Current membership of board or college for appropriate continuing education
2. Pre‐requisites
  * The physician must be independently privileged to perform the open/laparoscopic or minimally invasive procedures as applicable to the procedure for which he or she is requesting robotic assisted privileges.
  * Demonstrate performance of at least 20 open and laparoscopic surgeries with acceptable results and complications rates, reflective of the scope of privileges requested, in the past 12 months.
3. Provisional versus full credentialing
  To achieve credentialing there needs to be ‘sign‐off’, which can be viewed as two stages
  * Sign‐off by the mentor (for surgeon in practice) or
  * Fellowship/programme director (for fellow/resident) and
  * Sign‐off by a proctor.

Surgeons in practice, without robotic fellowship training, should be directed to follow the modular curriculum to acquire competency in a structured manner. They would need to identify a mentor to supervise this via component and then whole case completion prior to seeking a proctor to ‘sign‐off’.

- Procedure specific requirements:

Most mature credentialing frameworks require procedure specific credentialing. The NYU credentialing document requires each procedure (such as female pelvic floor, robotic cystectomy etc) to be assessed and signed off individually.^38^

- Maintaining proficiency. To continue with RAS, either basic or advanced procedures, the surgeon must perform a minimum 20 cases each calendar year using the robotic surgical system though it is unclear where the evidence for this number has been determined.^3^, ^36^, ^47^, ^48^ A study by Huffman et al critically reviewed robotic credentialling policies from 42 US institutions. 81% of the policies addressed Maintenance of Privilege (MOP) requirements with the recredentialing cycle running on a 12‐ or 24‐month basis. The average number of robotic cases accepted for MOP when adjusted to a 12‐month cycle was 7.19 ± 3.28 cases per year with a range of 1–15 cases per year.^48^ If less than 20 are performed, then the surgeon either needs to be supervised or demonstrate skills have been maintained with simulation training and cases should undergo ongoing focused review.^3^, ^36^, ^37^, ^38^
- Documenting Competency and Proficiency. Surgeons undertaking RAS should keep a record of total operative times, estimated blood loss and complications, with regular review by credentialling committee and/or individual maintaining simulation proficiency is also appropriate.^3^, ^36^, ^37^, ^38^
- Transferrable Credentialling. A surgeon with experience who is new to a given institution but has provided evidence of basic requirements should be provided independent credentials however the first 5 cases should be assisted by a robotic surgeon and undergo focused review by the credentialling committee or individual.^38^

Generally, the numbers required to be deemed competent are arbitrary. If a given trainee has competency in RARP with >20 supervised cases, it would follow that specific RFU credentialling may require fewer cases than where such prior credentialing exists.

## 6 ONGOING CREDENTIALLING REQUIREMENTS

Once credentialling is achieved, the next issues faced by institutions is how are they maintained. Generally, it is expected that a surgeon will continue to operate using RAS.

Though training has shifted towards competency‐based models, one of the shortcomings remains low case numbers/low volume centres. It is generally accepted that high case numbers do not necessarily equate to competency. A high volume of cases does however provide the opportunity to develop and apply skills and in turn demonstrate competency. Most credentialling requires completing a certain number of procedures, making it difficult to undertake at low volume centres. Given the uncommon nature of some operations, considerations need to be given to grouping procedures with similar skillsets to allow this to be achievable without significant impact on patient outcome. Proposed methods to maintain standards are annual simulation and regular undertaking of operative assistance to remain familiar with the docking and undocking of the robots and its equipment. Another means to assess surgical skill is surgeons should include audit of operative times, blood loss and complications based on Clavien–Dindo Classification.^49^ Lastly patient‐reported outcome measures could be considered to fill a vital gap in our perceived knowledge and expertise on outcomes that matter most to patients.

## 7 ADDRESSING CHALLENGES IN ROBOTIC FUNCTIONAL AND RECONSTRUCTIVE UROLOGY

The variety and heterogeneity of cases, combined with low numbers of some types of cases presents a challenge that differs significantly from existing robotic uro‐oncologic and other specialty procedures. Our group would like to identify a RFU curriculum with:

- modular curriculum with ‘components’, which could encompass skills across several different procedures to address volume constraints
- emphasis on index procedures to establish transferable skills, where possible, such as Burch Colposuspension or sacrocolpopexy or Burch takedown?
- develop the use of mixed reality/simulation models to support the opportunities to perform component skills in a safe environment, especially for complex procedures.

## 8 CONCLUSION

**FIGURE 1:**
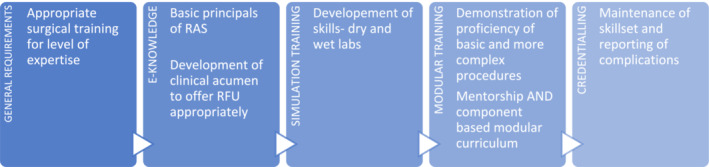
Proposed framework for robotic assisted surgery (RAS) training in functional and reconstructive urology.

Our group would like to use the above experiences to develop a guideline on training and credentialing of robotics in reconstruction and functional urology through the use of a modified Delphi study engaging specific societies and international expertise. The challenge in developing such a framework (Figure 1) in RFU is the relatively low case numbers and the low numbers of subspecialised surgeons. The use of reporting critical operative variables and complications provides a more standardised approach and would be more reflective on a surgeon's capabilities. Specifically, for RFU we would propose the addition of specific variables such as bowel, bladder, ureteric, vascular injury, fistula rates and chronic pain to the commonly reported operative time, blood loss and Clavien–Dindo complications. To overcome the low numbers, it will likely require a coordinated effort between sub‐speciality experts to develop the simulation and technology to ensure the appropriate proctorship is undertaken before credentialling.

## AUTHOR CONTRIBUTIONS

EF, HY, HH and HO created the initial concept of the work. FH wrote the initial manuscript. All authors refined the final manuscript, and agree to be accountable for all aspects of the work.

## DISCLOSURE OF INTERESTS

The authors declare that they have no disclosure of interest.
